# Depression among Chinese University Students: Prevalence and Socio-Demographic Correlates

**DOI:** 10.1371/journal.pone.0058379

**Published:** 2013-03-13

**Authors:** Lu Chen, Lin Wang, Xiao Hui Qiu, Xiu Xian Yang, Zheng Xue Qiao, Yan Jie Yang, Yuan Liang

**Affiliations:** 1 Clinical Department, Tong Ji Medical College, Hua Zhong University of Science and Technology, Wuhan, China; 2 Psychology Department, Public Health Institute, Harbin Medical University, Harbin China; Leicester University, United Kingdom

## Abstract

The purpose of the present study was to estimate the prevalence of depression in Chinese university students, and to identify the socio-demographic factors associated with depression in this population. A multi-stage stratified sampling procedure was used to select university students (N = 5245) in Harbin (Heilongjiang Province, Northeastern China), who were aged 16–35 years. The Beck Depression Inventory (BDI) was used to determine depressive symptoms of the participants. BDI scores of 14 or higher were categorized as depressive for logistic regression analysis. Depression was diagnosed by the Structured Clinical Interview (SCID) for the Diagnostic and Statistical Manual of Mental Disorders-Fourth Edition (DSM-IV). 11.7% of the participants had a BDI score 14 or higher. Major Depressive Disorder was seen in 4.0% of Chinese university students. There were no statistical differences in the incidence of depression when gender, ethnicity, and university classification were analyzed. Multivariate analysis showed that age, study year, satisfaction with major, family income situation, parental relationship and mother's education were significantly associated with depression. Moderate depression is prevalent in Chinese university students. The students who were older, dissatisfied with their major, had a lower family income, poor parental relationships, and a lower level of mother's education were susceptible to depression.

## Introduction 

Depressive disorder is one of the most common mental disorders, with lifetime prevalence of 16.2% and 12-month prevalence of 6.6% in the general population [1]. Depressive symptoms are widely distributed in the population and disrupt people's normal life. University students are a special group of people that are enduring a critical transitory period in which they are going from adolescence to adulthood and making many major life decisions. Previous studies reported that depressive symptoms in university students are noted around the world[2], [3], [4], [5] and the prevalence seems to be increasing [6]. Moreover, previous studies have shown that university students' health is poorer and there are higher rates of mental disorders, especially depression and anxiety, compared with their peers all over the world[7], [8], [9], [10], [11]. As a result, depression is a major, important problem and has been highlighted in university students, since depressive symptoms affect academic performance, are related to health, and may in extreme cases lead to suicide[12], [13]. In this crucial stage, university students can face many problems, such as accommodation, interpersonal relationships, competition and difficulties in academic studies, economic stress, and struggles with making important decisions. In addition, depression causes a significant burden to society and also is a disabling disorder that greatly limits everyday activity and productivity [14], [15], [16], [17], [18].

Recently there have been a number of studies of the prevalence of depression in university students worldwide [4], [19], [20], [21], [22], [23]. However, different studies used different assessments. Studies using the Beck Depression Inventory (BDI) [4], [21], PHQ-9 (a nine-item instrument based on the Diagnostic and Statistical Manual for Mental Disorder) [19], or the 42-item Depression Anxiety and Stress Scale (DASS-42)[7] can be found, and a minority of studies employed clear diagnostic criteria [22], [23], [24]. As a result, different conclusions are made by different criteria. Depressive symptoms in this study were assessed with the 21-item BDI. After the screening, we select the structured interview (SCID-I/P for DSM-IV) as the methods of diagnosing depression in students which was also used to make diagnose in students in previous studies[25]. The diagnosis of depression was made according to the DSM-IV classification system using the SCID. The severity of the depressive symptoms was measured using the Beck Depression Inventory (BDI)[26].

It is important to study depression among university students because most lifetime mental disorders have their first onset during the typical university age [27], and the mental health of university students has major implications for campus health services and mental health policy making [6], [28].

Our three purposes in this study are (a) to describe the prevalence of depression among a representative sample of Chinese university students, (b) to compare the prevalence of depressive symptoms and incidence of Major Depressive Disorder in our sample with that of previous studies, (c) to examine the socio-demographic correlates of depression in Chinese university students.

## Methods

### Study population

The study was conducted in Harbin, which is the capital of the Heilongjiang Province in Northeastern China. It lies on the southern bank of the Songhua River. Harbin is ranked as the tenth largest city in China, serving as a key political, economic, scientific, cultural and communications center of Northeastern China.

There are two main types of universities in China: one is the national key university and the other is the general institutions. The national key university receives a high level of support from the central government of the People's Republic of China. The general institutions are administered by the province. The type of university attended can decide your worth and social position. The students in the national key university face more stress from academic performance compared with students in the general institutions. There are a total of 14 universities in Harbin, three of them are the national key universities and 11 are ordinary universities. University students come to Harbin from throughout the country.

### Sample size and sampling technique

In order to get a representative sample of university students in China, we first chose two national key universities and four ordinary universities pragmatically by random numbers: Harbin Institute of Technology, Harbin Engineering University, Harbin Medical University, Harbin Normal University, Heilongjiang University, and Heilongjiang Institute of Technology, respectively. We then calculated the distribution of samples from every university by the proportion of students who attend the two main types of universities. We employed a stratified two-stage cluster selection of university students in full-time studies during the 2007/2008 academic year. We stratified the sample into five grades (first year, second year, third year, forth year, postgraduate year) and randomly selected classes from these grades. All students from the selected classes were invited to participate in the study. Thus, we randomly selected 6,000 students from total of 274,041 students in Harbin.

### Procedure

Approval for the study was given by the Ethics Committee of Harbin Medical University, Education Committee of Heilongjiang province and the institutional review committee of each of the selected universities. We personally contacted the selected students through the university, told them of the nature, purposes, benefits and adverse effects of the study, and invited them to participate. We ensured them of confidentiality and answered all related questions they raised. All participants were recruited directly in their respective classrooms after the end of a class. They were asked to make 15 min available for completion of the questionnaires. Participation was completely voluntary, with no economic or other motivation, and each participant signed written informed consent for their participation. The time of investigation avoided the beginning and end of the semester, when students are undergoing stressors related to moving or preparing for final exams and projects.

Depressive symptoms in this study were assessed with the 21-item BDI [29]. The BDI, created by Dr. Aaron T. Beck, is a self-report inventory, and one of the most widely used instruments for measuring the severity of depression. It can be used as a depression screening in non-clinical people. The BDI is a reliable and valid instrument[30]. Each statement in this inventory has a possible score range of 0 to 3, with the total score being 63. A score of 0 to 4 is considered as normal, 5 to 13 border line clinical depression, 14 to 20 moderate depression, and 21 to 63 severe depression. The internal consistency (Cronbach α) was high in many countries, ranging from 0.75 to 0.88[30]; in this study the Cronbach α is 0.851. The cut-off score for depression in this study was 14, as has been chosen in -several previous studies [31], [32]. BDI scores 14 or higher were categorized as depressive for logistic regression analysis. After the screening, a structured interview (SCID-I/P for DSM-IV) [33] was used to make diagnoses. The Structured Clinical Interview Axis I Disorders, Patient Version (SCID-I/P) were applied, and it was applied by face-to-face methods. A team of 15 psychiatrists were recruited and trained to make diagnoses.

The demographic variables included gender, age (16 to 43 years), ethnicity, major of study, year of study, satisfaction with major, parental relationship, maternal education and paternal level, university classification, and family economic situation. We defined <800 ¥as low income (poor), 800–2000 ¥ as middle income (moderate) and >2000 ¥ as high income (good), which is the criteria used by the State Labor and Social Security Department.

### Data analysis

The statistical package for social science 13.0 (SPSS 13.0) program was used for statistic analysis. All tests were 2-tailed, significance level was set at <0.05. Both undergraduate and postgraduate students were included in the analyses. We used logistic regression to assess the relationship between depressive symptoms and potential risk factors. The results are reported as odds ratios (OR) with 95% confidence interval (CI).

## Results

### Socio-demographic data of the participates

We distributed 6,000 questionnaires and 5,479 questionnaires were returned (91.3%). 521 potential participants declined to take part in the study. Excluding 234 invalid questionnaires (those with >20% questions unanswered), 5,245 students completed the screening questionnaire during the survey, with an 87.4% completion rate. Of these, 2,563 (48.9%) were male and 2,682 (51.1%) were female, 1,567 (29.9%) were from the national key universities and 3,678 (70.1%) were from the ordinary universities. There were 4,878 (93.0%) of the Han nationality, and 367 (7.0%) of other nationality. The average age of the participants in years was 21.3 (SD = 2.2), with a range of 16 to 43 years. Their families' economic situations were as follows: 354 (6.8%) good, 3,542 (67.5%) moderate, and 1,349 (25.7%) poor. Their parental relationships were as follows: 4,229 (80.6%) good, 785 (14.9%) moderate and 231 (4.5%) poor. Regarding their parents' educational situation, the distribution of the students was as follows for the highest level of education attained by the father: 2.9% uneducated, 17.4% primary school, 61.8% secondary or high school, and 17.9% university education. For the mother's education the highest level attained was as follows: 0.7% were uneducated, 11.9% primary school, 64.1% secondary or high school, and 23.3% university education. Regarding their major, the distribution of the students was as follows: 790 (15.1%), 662 (12.6%) management and 419 (7.9%) other major. Table 1 presents the socio-demographic factors of the sample.

**Table 1:** Socio-demographic factors of the sample in China university students (n = 5245).

Variables	Total	Depressed	Non-depressed	χ^2^	Df	*P*
Age						
16–25	5059	586	4473	4.466	1	0.035
25–35	186	31	155			
Gender						
Male	2563	306	2254	0.413	1	0.520
Female	2682	308	2374			
Ethnic						
Han	4878	568	4310	1.312	1	0.252
Other	367	50	317			
Family economic situation						
Poor	1349	226	1123	44.794	2	0.000
Moderate	3542	363	3179			
Good	354	29	325			
Study year						
1^st^	1572	190	1382	2.292	4	0.682
2^nd^	1294	160	1134			
3^rd^	1275	151	1124			
4^th^	717	75	642			
Postgraduate	387	41	346			
Major						
Science	790	122	668	26.206	5	0.000
Engineering	2409	265	2144			
Medicine	579	50	529			
Literature	386	62	324			
Management	662	66	596			
Others	419	52	367			
Parent relationships						
Good	4229	429	3800	59.940	3	0.000
Moderate	785	140	645			
Poor	231	47	184			
Mother education						
Uneducated0 year	36	9	27	59.940	3	0.000
Primary1–6 years	623	97	526			
Secondary or high7–12 years	123	25	98			
University13–25 years	108	22	86			
Father education						
Uneducated0 year	152	26	126	27.057	3	0.000
Primary1-6 years	914	147	2872			
Secondary or high7–12 years	3240	368	2872			
University13–25 years	939	76	863			
Satisfaction with major						
Yes	2109	160	1949	97.670	2	0.000
Moderate	2782	370	2412			
No	354	87	267			
University classification						
The national key university	1567	176	1391	0.604	1	0.437
The general institutions	3678	441	3237			

### Prevalence of depressive symptoms and depressive disorders

The mean BDI score was 6.31 (SD = 6.1), with a range between 0 and 63. Among them, 40.1% of participants had a score between 5 and 13 (i.e., borderline clinical depression); 8.4%, a score between 14 and 20 (i.e., moderate depression); and 3.3%, a score between 21 and 63 (i.e., severe depression). We learned form the previous studies that incidence of depression in 22.91% to 4.53%[34]. So we assume that the incidence of depression is 10% in Chinese university students. Firstly we randomly selected 210 students from the students whose BDI score was 13 or lower, and after the screening a structured interview (SCID-I/P for DSM-IV) was used to make diagnoses. On the basis of SCID interviews, we diagnosed nobody of the sample of whose BDI score between 5 and 13 as experiencing major depressive disorder. Out of all participants, 11.7% (n = 617) had a BDI score 14 or higher. Of these 617 participants, 139 refused to participate in the diagnostic interviews. At the end, 478 students participated in the interviews and one did not finish the interviews. On the basis of SCID interviews, we diagnosed 4.0% (n = 19, 19/477) of the sample as experiencing Major Depressive Disorder.

### Correlates of depressive symptoms

We found no significant association of depressive symptom with gender, ethnicity, year of study, or university classification (see Table 1). On the basis of a bivariate logistic model, the possibility of having depressive symptoms was significantly higher in students who were older than 25 years (OR = 1.792, 95% CI, 1.173–2.738), had a low family economic situation (OR = 1.339, 95% CI, 1.131–1.584), had a low level of education for the mother (OR = 1.286, 95% CI, 1.130–1.463), were dissatisfied with their major degree of study (OR = 1.894, 95%CI, 1.643–2.184), had poor parental relationships (OR = 1.410, 95% CI, 1.253–1.587), and was significantly higher in students who were in lower study year (OR = 0.930, 95% CI, 0.865–1.000) (see Table 2).

**Table 2:** Multilevel logistic regression on depression.

Variables	β	Waldχ^2^	P	OR	OR (95%CI)
Age	0.583	7.277	0.007	1.792	1.173–2.738
Study year	−0.073	3.883	0.049	0.930	0.865–1.000
Satisfaction with major	0.639	77.429	0.000	1.894	1.643–2.184
Family income situation	0.292	11.547	0.001	1.339	1.131–1.584
Parent relationships	0.344	32.597	0.000	1.410	1.253–1.587
Mother education	0.251	14.475	0.000	1.286	1.130–1.463

## Discussion

The present study is one of largest epidemiological studies about depression among Chinese university students. The study showed that the prevalence of depressive symptoms was 11.7%, and we diagnosed 4.0% of the sample as experiencing the Major Depressive Disorder. Steptoe et al. showed that Asian countries had the highest level depressive symptoms[21], which was not consistent with our result. The incidence of depressive symptoms and Major Depressive Disorder in our study were both lower than in other studies, such as Bayram & Bilgel reported that depression levels of moderate severity or above were found in 27.1% of Turkish university students, Bostanci reported out of all university students in Denizli, 26.2% had a BDI score 17 or higher[2], [4], [7]. However, depressive symptoms are slightly higher than Nair's study which reported 3% among school/college students had severe and extreme grades of depression [20]. These differences originated from different region, different measurement tools, different methods and different appraisal standards. University is an important transient life stage, with special academic, financial, and interpersonal pressures. Undergoing these transitions may lead to an increased risk of depression. However, the prevalence of depressive symptoms in the present study is a common incidence rate, similar to that seen in average people.

We found no differences in depressive symptoms between genders in our study. Similar to our results, some previous studies [2], [7], [35] showed that no differences in depressive symptoms were observed among male and female students. This might originate from the fact that Chinese female university students have equal political rights, experience the same pressure, and face same job opportunities as their male peers. However, some studies findings are contrary to our results, and found higher levels of depression among female students [4], [36], [37].

We found that older students were susceptible to depression compared with younger students. This may be because the older students face more stressful events than the younger students, such as employment, economic, graduation, and marriage pressures. Contrary to our study, some studies showed that older students reported lower levels of depression [21], [38].

We found little association between depressive symptoms and study year. Several studies showed that senior students had higher depression scores compared with freshmen [2], [39], similar to our result. However, some studies showed that first- and second-year students have higher depression scores compared with those in the higher years [7], [9], [40]. Dogan et al. found no difference in study year in terms of depression [41]. These differences in findings might be explained by different measurement tools or sample errors. In recent years, college enrollment in China has expanded consistently. The workload and employment pressure of Chinese students are increasing. Many undergraduate students prepare for the postgraduate entrance exam just as they enter the university. Postgraduate students worry about employment from the beginning of their graduate life. These stresses could lead to the occurrence of depressive symptoms.

In the present study, we found that there was the significant association between the satisfaction with the major and depression. Students who were satisfied with their major had lower depression scores than those who were not satisfied. In China, students' major generally is determined by their parents. When a student doesn′t like his major, they have lower interest, passion and learning motivation in academic performance. Thus the dissatisfaction with the major could result in depressive symptoms. The same results were obtained from the study by Bayram et al. (7Bayram & Bilgel, 2008).

We found that students from poor families had higher depression than those from well-off families. Similar results were obtained by many other studies [2], [7], [19], [21]. Lorant et al. carried out a meta-analysis to evaluate the relationship between socioeconomic status and depression. Results indicated that persons in the lowest socioeconomic group have 1.81 the likelihood of depression compared with those in the highest socioeconomic group [42]. Before Chinese economic reform, people's mental health was weakly influenced by people's economic status. This was because incomes were basically the same for all individuals. However, in recent years, China is in a transition period from a planned economy to a market economy, and it is now the case that individuals are given more pay for more work. It is increasingly the case that people's mental health is influenced by the economic conditions. This might be because economic conditions affect people's self-esteem and self-confidence. Low levels of self-esteem and self-confidence would lead to depressive symptoms.

A peculiar finding from our study was there was a negative correlation between the poor parental relationship and depressive symptoms. This result shows that the family environment affects students' emotional state. Parental relationships are the most important component in the family environment. China's special national conditions determine that there is only one child in one Chinese family. Most of parents try their best to love the only child. The poor relationship between parents could reduce the care of the child. The child's mental health is greatly influenced by the family environment.

Our study also found students with lower paternal education had higher depression. The educational level of parents is closely related to the students' mental health. Wu et al. found similar results in 2007[43]. Parents with higher level of education were able to pay close attention to students' psychological condition and actively communicate with students, which can increase students' psychological support. This is especially the case with the mother, as she is their children's first teacher and accompanies children more. The mother has a stronger influence over the children than father in China. A child could learn many behavior patterns from parents and be unconsciously influenced by parents' coping skills.

### Limitations

There were several limitations in the present study. The first limitation is the potential for sampling bias, as the sample in the study was only randomly selected from universities of Harbin. It is hard to generalize our findings to all Chinese students. However, we had a moderate sample size and used SCID interview to diagnose major depressive disorder. The second point is we only surveyed the depression distribution in university students and did not consider other psychological problems, such as anxiety. And the end sample size is small, further longitudinal, more accurate investigations are needed in order to confirm this merely preliminary report. However, the strategy of two step procedure of investigating the recognition of each specific diagnosed major depressive disorder that we used in our study should be improved. Another limitation of our study is that we had not considered family history of depression, stressful events, and social support. A future study may be required to examine the factors related to the genetic and environment variables.

## Conclusion

Moderate symptoms of depression appear in Chinese university students. The students who were older, dissatisfied with their major, had a lower family income, had a poor parental relationship, and lower level of education for their mother were susceptible to be depressed.
