# Correction: Depression among Chinese University Students: Prevalence and Socio-Demographic Correlates

**DOI:** 10.1371/annotation/e6648eb3-37d6-44d7-8052-979af14fa921

**Published:** 2013-11-06

**Authors:** Lu Chen, Lin Wang, Xiao Hui Qiu, Xiu Xian Yang, Zheng Xue Qiao, Yan Jie Yang, Yuan Liang

The correct affiliation for author Yuan Liang is: Department of Social Medicine, Public Health School, Tongji Medical College, Huazhong University of Science and Technology, Wuhan, Hubei, China

The correct affiliation for author Lin Wang is: Psychology Department of the Public Health Institute of Harbin Medical University, Harbin, China 

